# An investigation of *Mycobacterium bovis* and helminth coinfection in the European badger Meles meles

**DOI:** 10.1016/j.ijppaw.2022.11.001

**Published:** 2022-11-10

**Authors:** David J. Kelly, Nicola M. Marples, Rachel L. Byrne, Ursula Fogarty, Kevin Kenny, Henrietta Cameron, Denise Griffin, Celia V. Holland

**Affiliations:** a Department of Zoology, School of Natural Sciences, Trinity College Dublin, Ireland; b Tropical Disease Biology, Liverpool School of Tropical Medicine, Liverpool, UK; c Irish Equine Centre, Johnstown, Naas, Kildare, Ireland; d Central Veterinary Research Laboratory, Backweston, Kildare, Ireland

## Abstract

We investigated the relationship between the presence of helminth parasites in European badgers, and their tuberculosis (TB) status, culled as part of the bovine TB eradication programme in Ireland. Data on the worm burden or faecal egg or larval count was available for all helminth taxa recorded. Lymph node tissue samples were taken from the badgers and tested for TB. We then explored the correlation, in full-grown badgers, between the likelihood of *M. bovis* infection and both the prevalence and burden of certain helminth species. Specifically, our analyses focused upon the gastrointestinal species, *Uncinaria criniformis* and *Strongyloides* spp. We found that male badgers were more likely to have TB than female badgers, and that badgers infected with *U. criniformis* or *Strongyloides* spp. were more likely to have TB than badgers without such helminth infections. There was a suggestion that badgers with higher *U. criniformis* worm burdens were more likely to have TB than those with lesser burdens. Although our sampling protocols did not allow us to determine which infection came first, it strongly suggests that once badgers are infected with either gastrointestinal helminths or TB, they are likely to become coinfected. As Ireland works towards a national TB-free status, it will be important to appreciate the implications of such coinfection.

## Highlights

• European badgers are susceptible to both bacterial and helminth infections.
• Coinfection is more likely once either infection is contracted.
• The prevalence of gut helminths, but not their abundance, predicts TB infection.
• Male badgers are more likely to contract TB than female badgers.
• These findings have implications for the management of TB in wildlife.

## Graphical abstract

**Figure:**
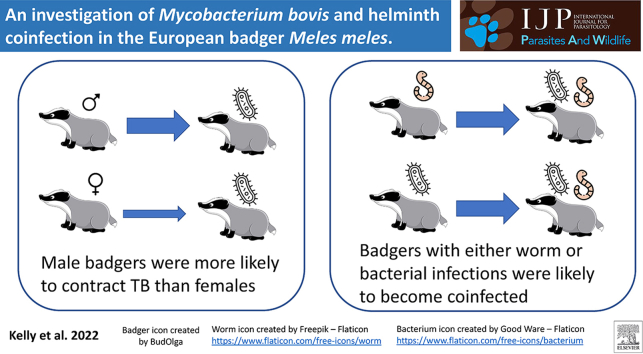

## 1 Introduction

Co-infection is now recognised to be a ubiquitous phenomenon with both humans and other animal hosts harbouring viral, bacterial, protist and helminth infections (Vaumourin et al., 2015). However, understanding the interrelationships and consequences of co-infections remains more challenging (Abbate et al., 2018). One aspect of co-infection that has received particular attention is the relationship between helminth infections and microparasites, such as malaria, HIV and TB (Massey et al., 2009; Ezenwa et al., 2010; Nacher, 2011; Babu and Nutman, 2016; Obieglo et al., 2016; Abbate et al., 2018; Wait et al., 2020). It has been hypothesised that the predominantly T helper type 2 (Th2) immune responses to helminth infection may perturb the vertebrate host T helper type 1 (Th1) response to accompanying microparasite infections (Maizels and Yazdanbakhsh, 2003). However, the main focus of these studies has been on humans (Kirwan et al., 2009). In contrast, Ezenwa et al. (2010) explored the interaction between helminths and *Mycobacterium bovis*, the causative agent of TB in African buffalo (*Syncerus caffer*) and demonstrated that buffalo which were more resistant to helminth infection had weaker Th1 responses, and that anthelmintic treatment enhanced Th1 immunity. In Europe, such an interaction between helminth parasites and tuberculosis could readily be investigated in the European badger (*Meles meles*).

European badgers are medium sized fossorial mammals, commonly living in agricultural areas near cattle farms. At higher densities t hey are territorial (Palphramand et al., 2007), live in social groups and defend their territories with active fighting, which is effective in spreading tuberculosis between individuals (Corner et al., 2011). Badgers regularly range outside their territories both for mating (Kelly et al., 2020) and while dispersing (Gaughran et al., 2019). In addition, some males hold territories comprising more than one social group (Gaughran et al., 2018). All this movement between territories is likely to facilitate the spread of infections across the badger population. Badgers forage on a wide range of food items (Cleary et al., 2009) including many species of soil-dwelling invertebrates, sometimes found by turning over mature cow-pats (Kruuk et al., 1979). As a result of these feeding habits, badgers encounter pathogens carried by cattle, including *M. bovis* (Barbier et al., 2016).

Badgers are a known maintenance host of *M. bovis* in Ireland and the UK (Gortazar et al., 2012). It is *M. bovis* which is the major cause of tuberculosis (TB) in cattle (OIE, 2019). Badgers have been identified as the source of *M. bovis* infections on farms in both the UK and Ireland (Ó Máirtín et al., 1998; McCulloch and Reiss, 2017). While it is now thought that most disease transfer is indirect (Mullen et al., 2013; O'Mahony, 2014; Woodroffe et al., 2016), the evidence that European badgers and cattle share local strains of *M. bovis* is incontrovertible (Goodchild et al., 2012; Payne et al., 2013; Barron et al., 2018; Milne et al., 2020).

In addition to their importance in the transmission of TB, badgers act as hosts for a wide range of parasites (Hancox, 1980). A number of subsequent studies have considered the helminth parasites of European badgers, although few have recorded the intensity of infection or the diversity of the helminth parasite community. Torres et al. (2001) described a rich helminth parasite fauna (17 species) from 85 European badgers sampled from Spain, including cestodes, trematodes and nematodes. In a sample of 289 European badgers sampled from Ireland, from which abundance and intensity was assessed by means of both worm counts and faecal egg or larval counts, eight distinct helminth taxa were described (Byrne et al., 2020). Of particular note was infection with the hookworm species, *Uncinaria criniformis*, which exhibited the highest prevalence among all the nematode taxa detected. This hookworm showed a highly aggregated distribution and some badgers harboured very heavy worm burdens (Byrne et al., 2018, 2020). In contrast to the Spanish study, all helminths recorded in the Irish study were nematodes.

There is a current, and increasing, interest in co-infection and its potential effects on vaccination (Wait et al., 2020). However, to our knowledge, only a single study on the relationship between helminth parasitism and TB in badgers has been published (Massey et al., 2009). This relatively small study (28 badgers) described 14 species of protist and helminth parasites, detected by faecal analysis. No relationship between TB status and the diversity or intensity of parasitic infection was found. The authors concluded that studies with larger sample sizes were required to confirm their observations.

Both England (Defra, 2014) and Ireland (DAFM, 2018) have stated their ambitions to be TB-free by the 2030s. The control of TB in wildlife populations is seen as a fundamental part of those ambitions (Good et al., 2018). Until vaccination programmes can be implemented at a national scale, the culling of badger populations remains a contentious, yet necessary, part of TB control (Martin et al., 2020). In Ireland, as a consequence, the efficacy of TB vaccination is vital for both conservation of badgers, and the eradication of bovine TB. However, such efficacy may be undermined by co-infections with helminths. Buddle et al. (2018) state: “Possible reasons for the failure [of BCG vaccine] to protect [cattle] in field trials could include … prior sensitisation to helminths.” We were unable to find data on the same scenario with badgers. In a mouse model (Obieglo et al., 2016), chronic gastrointestinal nematode infection mutes immune responses to mycobacterial infection. Both studies suggest there is some “perturbation” of the immune response to bacterial infection caused by the presence of helminth infection. In contrast to this apparent interaction of helminth and bacterial infections, the liver fluke appears to have little or no effect on TB test status in cattle (Byrne et al., 2019a; Howell et al., 2019).

Therefore, the aim of this study is to explore the relationship between TB status and helminth parasitism in a large sample of badgers for which detailed information on the prevalence (presence/absence) and abundance (worm burden or larval count) of the helminth parasites was known.

## 2 Methods

### 2.1 Study animals

In total, 268 full-grown badgers were examined for helminth parasites and assessed for TB status. Our badger cohort consisted of adults (n = 253) and juveniles (n = 15). Age was assessed by size, dentition and the development of the reproductive tract. Badgers were provided by the Irish Department of Agriculture, Food and the Marine (DAFM) from the strategic culling conducted in bovine TB endemic regions by DAFM, under a National Parks and Wildlife Services license. No animals were killed specifically for the purposes of this study.

Badgers were assigned unique identification codes, euthanised and transported from their site of capture to the laboratory for post mortem examination within 1–3 days and dissected on the day of arrival. The gross post mortem examination involved inspection of the surface of the badger, the thoracic and abdominal cavities, together with all of the organs present and the lymph nodes. The following lymph nodes were collected and pooled for bacteriological examination: submandibular, parotid, retropharyngeal, prescapular, axillary, external inguinal, bronchial and mediastinal and immediately frozen. Any gross lesions noted on gross post mortem examination were also collected for culture. Badgers for post mortem examination were chosen at random from Western counties and Eastern counties across one sample year (Byrne et al., 2020). Laboratory work at Trinity College Dublin was approved by the School of Natural Sciences Research Ethics Committee (code: 2016–28).

### 2.2 Mycobacterial culture

The frozen pooled tissue samples were thawed overnight, placed in a stomacher bag containing 20 mL of saline and homogenised. Ten millilitres of homogenate were transferred to a sterile polypropylene tube and decontaminated using oxalic acid at a final concentration of 5%. After centrifugation and rinse steps (Mehra and Philips, 2014), the resultant pellet was resuspended in 1 ml of sterile phosphate buffered saline. One 7 ml slope of Lowenstein Jensen media and one 7 ml slope of modified 7H11 medium, each containing sodium pyruvate, were each inoculated with 0.2 ml of the suspension (Gormley et al., 2021). A BD BACTEC (Becton Dickinson and Company, NJ, USA) Mycobacterial Growth Indicator Tube (MGIT) received 0.5 ml of the inoculum. Solid media were incubated at 37 °C for 7 weeks, examined for growth each week and smears were prepared from suspect colonies for staining by the Ziehl-Neelsen method (Christie and Callihan, 1995). MGIT tubes were incubated for 7 weeks in the BD BACTEC instrument and smears were prepared from tubes which displayed a growth signal.

Media containing acid-fast bacilli were sampled for PCR testing. A suspension of mycobacteria from solid or liquid media was heat killed and DNA extracted using an InstaGene™ matrix (BioRad, Hercules, CA, USA). This DNA was tested in a real-time multiplex PCR for the presence or absence of RD1, RD4 and a conserved region external to RD9 (Halse et al., 2011), to allow identification to the species level within the *Mycobacterium tuberculosis* complex. The multiplex PCR also included a target for members of the *Mycobacterium* genus (Torvinen et al., 2010). The PCR was run for 35 cycles and samples which displayed fluorescence greater than the threshold value for this cycle number were classed as positive for a given target. Isolates were identified accordingly as *M. bovis* or non-tuberculous mycobacteria.

We are aware the prevalence of *M. bovis* in this study population is probably an underestimate, since all available protocols to maximise its detection could not be followed. Sampling of additional lymph nodes during post mortem examination (Murphy et al., 2010; Corner et al., 2012a), culturing from individual lymph nodes, rather than pooling (assumed to dilute any mycobacteria present), and the extended incubation of samples (Corner et al., 2012b) may all have increased the detection of *M. bovis*. Despite these limitations, we estimate that our protocol detected ∼90% of animals infected with *M. bovis*.

### 2.3 Collection and processing of samples for parasitological analysis

Lungs and gastrointestinal (GI) tracts were collected and frozen without preservative, at −20 °C (for 3–12 months) until examination. Organs were defrosted overnight at 5 °C and inspected by dissection during the next day. The lungs were processed and examined by a modified flush and dissection technique (Byrne et al., 2018). GI tracts were cut open lengthways, flushed using 0.9% saline and examined by eye for helminth parasites. Any helminth parasites detected were removed, counted and preserved in 70% ethanol for subsequent identification (Byrne et al., 2020). Species identification was confirmed by parasitologist, Eileen Harris, at the Natural History Museum, London.

Faecal samples were extruded from the rectum and fixed in 10% formalin. A modified formol-ether concentration technique (Allen and Ridley, 1970) was used to detect helminth eggs and larvae in faecal samples. Samples were processed blind (i.e. without knowledge of the outcome of GI analysis) with two slides examined for each individual badger and the egg per gram count and larvae per gram calculated for each sample.

### 2.4 Parasitological parameters

The analyses presented were based upon a sample of 268 full-grown badgers for which TB status was assessed. After initial analysis, the gastrointestinal species *Uncinaria criniformis* and *Strongyloides* spp. were selected in order to explore the relationship between helminth parasitism and TB status. These species had the highest prevalences within the original parasite community analysis (Byrne et al., 2020). The prevalence and abundance of each helminth species was calculated based upon worm burden data in the case of *Uncinaria criniformis* or faecal larval counts in the case of *Strongyloides* spp.

### 2.5 Statistical Methods

Analyses were performed in R (R Core Team, 2020). To investigate the correlation between worm infection and TB infection, we used the *glmm* function from the **glmm** package (Knudson, 2020) to run Generalized Linear Mixed Models (GLMMs) with a binomial distribution. The *glmm* package uses a Monte Carlo likelihood approximation (MCLA) to estimate the likelihood function, which facilitates the reporting of P values for the fixed and random effects. We maximised the Monte Carlo sample size (*m*) in order to produce a more accurate MCLA (Knudson et al., 2021), which, in turn, produced more accurate Monte Carlo maximum likelihood estimates (MCMLEs). We checked the Monte Carlo standard errors (MCSEs) to decide whether a particular Monte Carlo sample size was large enough. Each MCSE should be small compared to its corresponding SE (Knudson et al., 2021). In line with this restriction, we required all MCSEs to be <10% of their corresponding SEs.

Our GLMMs predicted TB status (positive or negative for *M. bovis* isolation – see above) using worm prevalence (i.e. present/absent), the sex of the badger, and the background rate of TB in cattle in the county where the sett (the social group's underground network of tunnels and chambers) was located (Kelly et al., 2021). We ran several models with different worm cohorts (models A1, A2 and A3), to investigate whether the presence of some worm species were better than others at predicting the TB status of badgers.

Having identified the best model for predicting a correlation between helminth infection and TB infection, we conducted further analyses to investigate the correlation between worm burden (the number of adult worms present in a worm-infected badger) and TB infection. Initially, this was run as a GLMM (model B1) to avoid the simple pseudoreplication (Hurlbert, 1984) of multiple badgers from the same sett. However, it appeared the inclusion of sett id as a random factor over exaggerated the importance of multiple badgers from certain setts. Since relatively few setts contained multiple badgers (only 31 of the 225 setts in the study), we adopted another approach. We used the *glm* function (part of the basic R environment) to run Generalized Linear Models (GLMs) with a binomial distribution, limiting the dataset to one badger from any given sett. To prepare the data for GLM analysis, we drew a single badger, randomly, from each sett which contained multiple badgers. We then ran three GLMs using this dataset (models C1, C2 and C3). In order to minimise any bias in the sampling, we repeated the random draw process 1000 times. Our findings for this analysis were based on a summary of these 1000 GLMs. Our GLMs used TB status (positive or negative for *M. bovis* isolation – see above) as the dependent variable and worm burden, badger sex and the background rate of TB in the county where the sett was located (see Kelly et al. (2021)) as potential fixed factors.

The R code for all models is included in an appendix (Appendix A), for the reference of readers.

## 3 Results

Of the 268 full-grown badgers (138 males and 130 females) from the Republic of Ireland, 16.4% were positive for TB. The prevalence, mean abundance, and the range (minimum to maximum) of helminth infection are shown in Table 1. All helminth taxa identified were nematodes and the helminth parasite community was dominated by *U. criniformis*, a hookworm species, with a prevalence of 58.2% and a mean worm burden of 22 worms per badger (Table 1). Furthermore, with a variance to mean ratio of 128, the distribution of hookworm among the badgers was highly aggregated, with some individuals harbouring very high worm burdens (Byrne et al., 2018).

**Table 1:** The prevalence, mean abundance (±S.E.) and range (minimum-maximum) of identified helminth parasites in adult European badgers (n = 268) collected in Ireland.

		Prevalence % (95% CI)	Mean Abundance ±S.E	min-max
Adult worm burden	Uncinaria criniformis	58.2	22.25 ± 3.3	0–500
		(52.1–64.2)		
	*Perostrongylus falciformis*^a^	27.2	2.19 ± 0.425	0–53
		(22.0–32.0)		
	*Crenosoma melesi*	1.12	0.88 ± 0.84	0–224
		(0.2–3.2)		
Faecal egg/larval counts	*Strongyloides* spp.	28.3	14.2 ± 2.9	0–540
		(23.0–34.2)		
	*Eucoleus aerophilus*	8.2	1.83 ± 0.56	0–90
		(5.2–12.2)		

a We follow the taxonomic revision of (Deak et al., 2018).

The second most prevalent species was *Strongyloides* spp. with a prevalence of 28.3% and a mean count of 14 larvae per host. Unfortunately, these parasites could not be identified to species as they were present in their larval stage.

We performed an initial GLMM (model A1 – code available in Appendix A) using the presence of any worm species to predict the TB status of badgers, along with information on the sex of the badger (M/F) and the background level of *M. bovis* infection in the county where the sett was located (Kelly et al., 2021), as fixed factors. We also included the sett identifier as a random factor (the data used for these analyses are provided in Appendix B). This model failed to identify any correlations (P values for all correlations >0.05). A second GLMM (model A2) included data from the three most prevalent worms (*Uncinaria*, *Strongyloides* and *Perostrongylus*) with the same additional fixed and random factors as model A1. This model also failed to identify any correlations (P values for all correlations >0.05). We then conducted a third GLMM (model A3) which considered only the prevalence of *Uncinaria* or *Strongyloides* (the gastrointestinal species) to predict the TB status of badgers (model A3). We included information on the sex of the badger (M/F) and the background level of *M. bovis* infection in the county where the sett was located, as fixed factors. We also included the sett identifier as a random factor (the data used for these models are given in Appendix B).

We found that male badgers were more likely to have TB than female badgers (effect of sex in Fixed Effects output – Table 2) and that badgers with hookworm or *Strongyloides* infections were more likely to have TB than those without such infections (effect of hook.or.strong in Fixed Effects output – Table 2). The background level of TB in the county was not correlated with the TB status of badgers. However, the inclusion of this term strengthened both of the correlations of the other fixed factors (sex and the presence of gastrointestinal nematodes) with the TB status of badgers.

**Table 2:** Output from GLMM (model A3) using a Monte Carlo sample size (m) of 1,000,000.

Fixed Effects:					
	Estimate	Std. Error	z value	Pr(>|z|)	
(Intercept)	−3.930	0.920	−4.280	1.87e-05	***
hook.or.strong	1.310	0.480	2.736	0.00622	**
sex	0.766	0.368	2.081	0.03740	*
local.TB.density	11.100	12.000	0.922	0.35647	

Random Effects (P-values are one-tailed):					
	Estimate	Std. Error	z value	Pr(>|z|)	
sett.num	0.625	0.070	8.98	<2e-16	***

As hookworm were identified by the presence of adult worms, it was possible to investigate whether a higher burden of hookworm was more likely to predict *M. bovis* infection in individual badgers. An initial GLMM analysis (model B1) failed to identify a correlation between *M. bovis* infection and worm burden (Monte Carlo GLMM, correlation with log(hookworm burden); estimate = 0.424, z = 1.714, P = 0.0866). We were uncertain GLMM was an appropriate analysis for this dataset, so we pursued a GLM approach, using one badger from any given sett (see Statistical Methods). However, as even a random draw of individual badgers from each sett might have biased our findings, we ran 1000 GLMs against randomly drawn collections of badgers for this analysis (Appendix C, summarised in Table 3).

**Table 3:** Summary of output from the 100 GLMs (Models C1, C2 and C3). The presence of dependent variables in the models is indicated by ticks (✓). In addition, the percentage of models which showed a correlation between worm burden and TB presence (worms ∼ TB), with a P value < 0.05, is also provided.

Model ID	worm burden	badger sex	background TB	worms ∼ TB
C1	✓	✓	✓	58%
C2	✓	✓		42%
C3	✓			23%

The output from these GLMs differed between unique, randomly-drawn datasets (Appendix C, summarised in Table 3). Overall, models with greater complexity (from C1, the most complex, to C3, the least complex) suggested a greater likelihood of correlation between hookworm burden and *M. bovis* infection. When using the saturated GLM (model C1 – Appendix A), we found that hookworm burden was correlated with TB status for 577 of the 1000 runs (58%). When the measure of background TB was excluded (model C2), we found that hookworm burden was correlated with TB status for 421 of the 1000 runs (42%). And when both the measure of background TB and the sex of the badger were excluded (model C3), we found that hookworm burden was correlated with TB status for only 231 of the 1000 runs (23%). These findings support the view that it is important to allow for the sex of the badgers sampled, as well as the local background rate of tuberculosis in the environment, when considering correlations between TB and gastrointestinal helminth infections in badgers. In accordance with the correlation of worm prevalence and *M. bovis* infection (model A3), these subsequent analyses (models C1, C2 and C3) suggest that badgers with higher hookworm burdens are more prone to TB.

## 4 Discussion

We demonstrate a link between gastrointestinal helminth infection and *M. bovis* infection in European badgers, utilising data from 289 badgers. Such a finding appears intuitive, supporting the idea that *M. bovis* infections and helminth infections trigger the antagonistic Th1 and Th2 immune pathways, respectively. The correlation of helminth and *M. bovis* infections was only evident when we limited our analyses to gastrointestinal helminths (model A3). It appears that helminth infections in other organs of the badger (e.g. *Perostrongylus* in the lungs), do not increase the likelihood of *M. bovis* infection (models A1 and A2) in the same way gastrointestinal helminth infections do.

We found it was important to account for the sex of the badger in our analyses and that male badgers were more likely to be infected with *M. bovis*. This may be linked to behaviour, as adult male badgers are more likely to show bite-wounding than adult females (Delahay et al., 2006; Gaughran et al., 2018), and biting is an efficient way of transmitting *M*. *bovis* infection (Corner et al., 2011). However, other studies have shown that male badgers are more likely to be infected with helminths than female badgers (Byrne et al., 2019b). In our models, it was also important to account for the local density of *M. bovis* infections. Badgers from setts in areas with higher local TB densities in cattle showed a greater tendency to be infected with TB than badgers from setts in areas with lower local TB densities. Although this was not a strong correlation, the inclusion of this term (local.TB.density) as a fixed factor contributed to the overall performance of our models.

We might expect animals with greater helminth burdens to show more highly stimulated Th2 pathways and therefore be more susceptible to microparasitic infections (e.g. *M. bovis*) (Maizels and Yazdanbakhsh, 2003). The majority of GLMs (model C3) performed against randomly-drawn datasets (58%) showed a correlation between hookworm burden and *M. bovis* infection, when also accounting for the sex of the badger and the local density of tuberculosis. The significance of the correlation between hookworm burden and *M. bovis* infection varied between 0.008 and 0.20 in those 1000 randomly-drawn GLMs (Appendix C). This variation clearly demonstrates the bias that the random selection of an individual badger from a sett may have on the outcome of a correlation under test. At the same time, the increase in the number of random draws which produced significant correlations between hookworm burden and *M. bovis* infection as the models increased in complexity (from C3 to C2 to C1) suggests future investigations may uncover a more direct relationship.

The prevalence of TB within our study population was low in absolute terms (44 of 269 badgers = 16.4%) and compared to a previous study in Ireland (43.2%) (Corner et al., 2012a), but relatively high compared to randomly sampled badger populations across the UK (Wales – 7.3% (Schroeder et al., 2021); England – 8.3% (Swift et al., 2021)). These differences reflect the reactive nature of the badger trapping which provided both us and Corner et al. (2012) with samples, i.e. the badgers were targeted in areas which were experiencing TB outbreaks (cf High TB Area East – 18.6% (Schroeder et al., 2021); highest county density – 15% (Swift et al., 2021)). The frequency distribution of *Strongyloides* spp. was aggregated (variance to mean ratio = 3.3), whereas *U. criniformis* was highly aggregated (variance to mean ratio = 128). Both the low prevalence of TB in our badger population and the aggregated nature of the helminth worms reduce the likelihood of detecting a correlation between the two. Therefore our finding of a strong correlation between the presence of worms and tuberculosis supports the view that the effect of one infection in promoting susceptibility to the other is biologically significant.

Unfortunately, as our study sampled each of the badgers at a single point in time, we were unable to determine a sequence to coinfection. Since both *M. bovis* and the infective stages of helminths can survive independently of their hosts in the environment (Fine et al., 2011; Barbier et al., 2017; Steinbaum et al., 2017), these infections may simply be contracted in the order in which badgers encounter them. However, once a badger is infected with *M. bovis*, it is likely that they will become infected with helminths, and vice versa.

In summary, the correlation between TB and gastrointestinal helminth infections in this population of badgers indicates that contracting one infection increases susceptibility to the other to a significant degree. This raises an important question. Might the efficacy of vaccination of badgers against TB be compromised by the presence of gastrointestinal helminth infections in the population? Since we know from previous work that the prevalence of helminths in Irish badgers is rather high (83% of the population - Byrne et al., 2019a, 2019b) it seems likely that any TB vaccination programme of badgers would be enhanced by a consideration of helminth infections within the population.

## Availability of data and materials

Data supporting the conclusions of this article are included in the article and its additional files. Raw data are available upon request to the first author.

## Declaration of competing interest

The authors report no conflicts of interests.
