# Supplementary material for: An investigation of Mycobacterium bovis and helminth coinfection in the European badger Meles meles
Source: Int J Parasitol Parasites Wildl. 2022 Nov 10;19:311–6. doi: 10.1016/j.ijppaw.2022.11.001 (PMC9700262; doi:10.1016/j.ijppaw.2022.11.001)
Supplement: Multimedia component 1 [file mmc1.docx]

**Appendix A. R code for the linear and mixed models used in our analyses.**

**Model A1.**

glmm(TB.status ~ worm.presence + badger.gender + local.TB.density, list(TB ~ 0 + sett.num),

varcomps.names=c("sett.num"), family.glmm=binomial.glmm,

m=1000000, debug=TRUE, data = badger.data)

**Model A2.**

glmm(TB.status ~ hook.or.strong.or.aeluro.presence + badger.gender + local.TB.density,

list(TB ~ 0 + sett.num), varcomps.names=c("sett.num"),

family.glmm=binomial.glmm, m=1000000, debug=TRUE, data = badger.data)

**Model A3.**

glmm(TB.status ~ hook.or.strong.presence + badger.gender + local.TB.density, list(TB ~ 0 +

sett.num), varcomps.names=c("sett.num"), family.glmm=binomial.glmm, m=1000000, debug=TRUE, data = badger.data)

**Model B1.**

glmm(TB.status ~ log(hookworm.count) + badger.gender + local.TB.density, list(TB ~ 0 +

sett.num), varcomps.names=c("sett.num"), family.glmm=binomial.glmm, m=1000000, debug=TRUE, data = badger.data)

**Model C1.**

glm(TB ~ log(hookworm.count) + badger.gender + local.TB.density,

family=binomial(link='logit'), na.action = "na.fail", data = badger.data)

**Model C2.**

glm(TB ~ log(hookworm.count) + badger.gender, family=binomial(link='logit'), na.action =

"na.fail", data = badger.data)

**Model C3.**

glm(TB ~ log(hookworm.count), family=binomial(link='logit'), na.action = "na.fail", data =

badger.data)
